# CD8^+^ T Cells Mediate the Athero-Protective Effect of Immunization with an ApoB-100 Peptide

**DOI:** 10.1371/journal.pone.0030780

**Published:** 2012-02-09

**Authors:** Kuang-Yuh Chyu, Xiaoning Zhao, Paul C. Dimayuga, Jianchang Zhou, Xiaojun Li, Juliana Yano, Wai Man Lio, Lai Fan Chan, Jonathan Kirzner, Portia Trinidad, Bojan Cercek, Prediman K. Shah

**Affiliations:** Division of Cardiology, Oppenheimer Atherosclerosis Research Center, Cedars-Sinai Heart Institute, Cedars-Sinai Medical Center, Los Angeles, California, United States of America; University of Palermo, Italy

## Abstract

Immunization of hypercholesterolemic mice with selected apoB-100 peptide antigens reduces atherosclerosis but the precise immune mediators of athero-protection remain unclear. In this study we show that immunization of apoE (-/-) mice with p210, a 20 amino acid apoB-100 related peptide, reduced aortic atherosclerosis compared with PBS or adjuvant/carrier controls. Immunization with p210 activated CD8^+^ T cells, reduced dendritic cells (DC) at the site of immunization and within the plaque with an associated reduction in plaque macrophage immunoreactivity. Adoptive transfer of CD8^+^ T cells from p210 immunized mice recapitulated the athero-protective effect of p210 immunization in naïve, non-immunized mice. CD8^+^ T cells from p210 immunized mice developed a preferentially higher cytolytic response against p210-loaded dendritic cells in vitro. Although p210 immunization profoundly modulated DCs and cellular immune responses, it did not alter the efficacy of subsequent T cell dependent or independent immune response to other irrelevant antigens. Our data define, for the first time, a role for CD8^+^ T cells in mediating the athero-protective effects of apoB-100 related peptide immunization in apoE (-/-) mice.

## Introduction

Adaptive and innate immunity have been implicated in atherogenesis and pre-clinical studies have suggested that immuno-modulating therapies can reduce atherosclerosis [1], [2]. One such strategy involves active immunization using apoB-100 related peptide antigens [3], [4].

Although active immunization using several different apoB-100 peptides reduces atherosclerosis [1], [3]–[5], the humoral or cellular immune mediators of such effect have not been fully elucidated. Recent reports show that different immunization strategies using the same peptide antigen (apoB-100 related peptide p210) yield different immune responses, yet still provide protection against atherosclerosis [6], [7]. Subcutaneous immunization of LDLR(-/-)/human apoB-100 transgenic mice with p210 did not elicit an increase of anti-p210 antibody response compared with carrier control but reduced atherosclerosis by 59% [6]. No specific mechanism was delineated in the report. On the other hand, intranasal immunization of apoE(-/-) mice with a p210-CTB fusion protein preparation reduced atherosclerosis by 35% with increased IgG titers against p210 and CD4^+^ T regulatory cells without further elucidation of the role of either immune response [7]. Nevertheless, both studies concluded that the protection against atherosclerosis was independent of p210 antibody response. Thus how the immune response to p210 immunization mediates protection against atherosclerosis still remains largely unknown.

In this study, we therefore designed a series of experiments to characterize the immune response to p210 immunization and to define the type of immune cells that mediate the athero-protective effect of p210 immunization.

## Results

### p210 immunization reduced atherosclerosis

**Figure 1:**
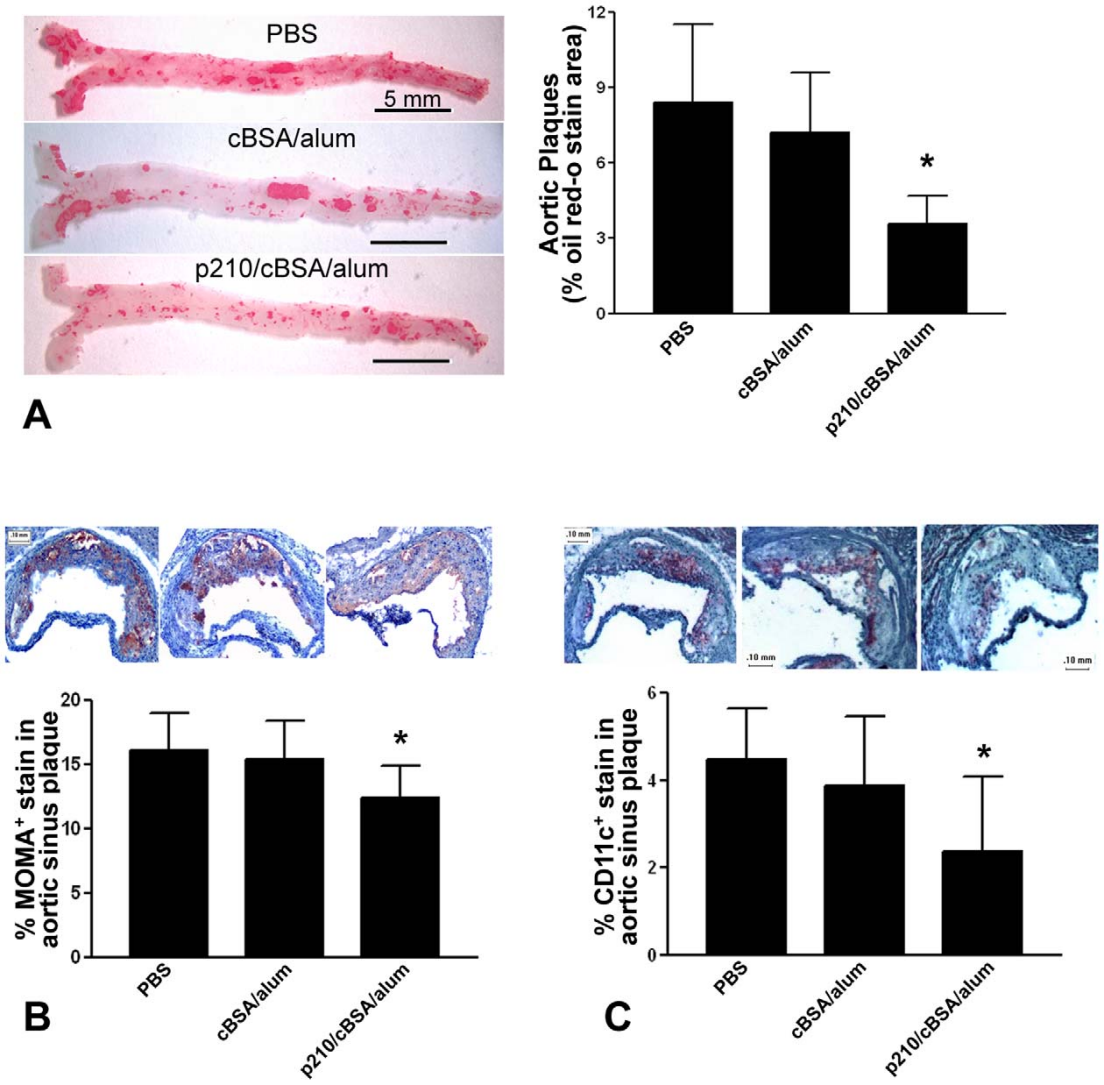
p210 immunization confers protection against atherosclerosis. Representative pictures of aortic en-face lipid staining from each group shown (A; left panel). Immunization with native p210 resulted in a significant reduction in aortic atherosclerosis when compared to PBS and cBSA/Alum group (A; right panel; n = 9–10 each group). P210 immunization significantly reduced macrophage infiltration (B) assessed by MOMA-2 stain (n = 9–10 each group) and DC presence (C) assessed by CD11c (n = 7–12 each group) stain in aortic sinus plaques. Positive stain indicated by reddish-brown color. Data were analyzed by ANOVA followed by Newman-Keuls multiple group comparison, *P<0.05 vs. PBS and cBSA/alum.

Immunization with p210 reduced aortic atherosclerosis by 57% and 50% compared to PBS and cBSA/Alum group, respectively (Fig. 1A) without significant difference in circulating cholesterol levels or body weight (Table 1). The aortic sinus plaques from p210/cBSA/alum group contained significantly reduced macrophage and dendritic cell (DC) immuno-reactivity assessed by MOMA-2 (Fig. 1B) and CD11c (Fig. 1C) immunohistochemical staining respectively with no difference in the aortic sinus lesion size (Table 1). There was no difference in CD4^+^ T cells, but a significant reduction in CD8^+^ T cells in both the cBSA/Alum group and the p210/cBSA/alum group compared to PBS (Table 1).

**Table 1:** Circulating level of cholesterol and body weight of mice from PBS, cBSA/alum and p210/cBSA/alum group at euthanasia.

	PBS	cBSA/alum	P210/cBSA/alum	P value (ANOVA)
**Cholesterol (mg/dl; N = 10)**	1503±485	1395±420	1135±381	NS
**Body weight (gm; N = 10)**	37.9±5.4	34.8±5.4	34.3±6.5	NS
**Aortic sinus plaque size (mm^2^; N = 10)**	0.40±0.13	0.42±0.09	0.40±0.08	NS
**Aortic sinus plaque CD4^+^ (% area; N≥5)**	0.21±0.21	0.17±0.15	0.36±0.26	NS
**Aortic sinus plaque CD8^+^ (% area; N≥5)**	0.52±0.35	0.11±0.25*	0.21±0.27*	0.03

*P<0.05 vs. PBS (post-hoc test).

### Characterization of response to p210 immunization

#### Antibody response to p210 immunization

**Figure 2:**
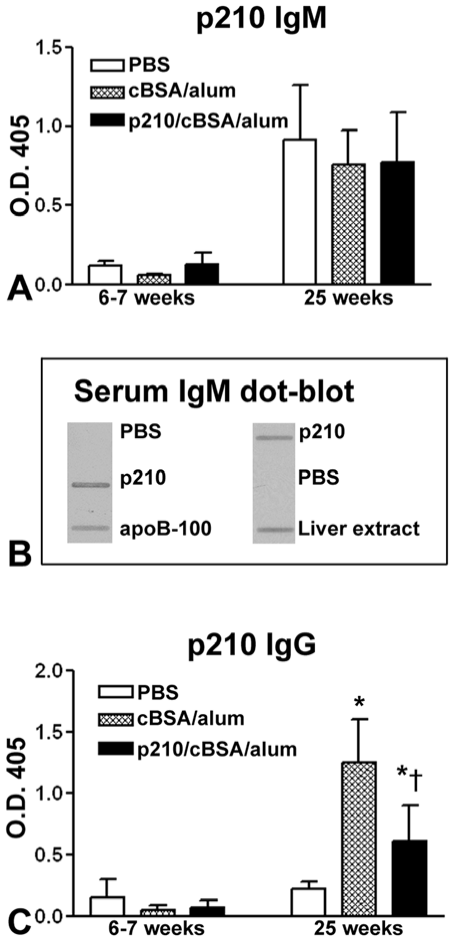
Antibody response to p210 immunization. (A) P210 IgM antibody levels were low before immunization and significantly increased at euthanasia with no difference among the 3 groups of mice. (B) Immune-reactivity of serum IgM from p210/cBSA/alum mice against dot blot of human apoB-100 and mouse liver protein extract. (C) P210 IgG antibody levels were low before immunization and remained low in the PBS group at euthanasia but significantly increased in cBSA/alum and p210/cBSA/alum groups, with the highest levels in the cBSA/alum group. N = 5 each for 6–7 week time-point and n = 9 each for 25 week time-point. Data were analyzed by ANOVA followed by Newman-Keuls multiple group comparison, *P<0.05 vs. PBS; †P<0.05 vs. cBSA/alum.

IgM levels against p210 were low in all groups prior to immunization at 6–7 weeks of age. There was a significant increase in p210 IgM titer in all groups at euthanasia at 25 weeks of age (Fig. 2A), suggesting an immune response against endogenous p210. To verify if p210 is indeed a self-antigen that elicits an antibody response, we performed dot-blot assays to test the reactivity of serum IgM from p210-immunized mice on purified human apoB-100 and mouse liver protein extract with p210 as positive and PBS as negative controls, respectively. Dot blots showed serum IgM reactivity to human apoB-100 and mouse liver extract (Fig. 2B).

IgG antibodies against p210 were low in all 3 groups of mice prior to immunization. At euthanasia at 25 weeks of age, there was a significant increase in p210 IgG antibodies in both cBSA/alum and p210/cBSA/alum groups compared with the PBS group, but levels in the cBSA/alum group was the highest between the 2 responding groups (Fig. 2C). The increase in IgG antibodies against p210 in both cBSA/alum group and p210/cBSA/alum but not in the PBS group suggests that immunoglobulin class switching occurred in response to the adjuvant.

#### CD4^+^ T cell response to immunization

**Figure 3:**
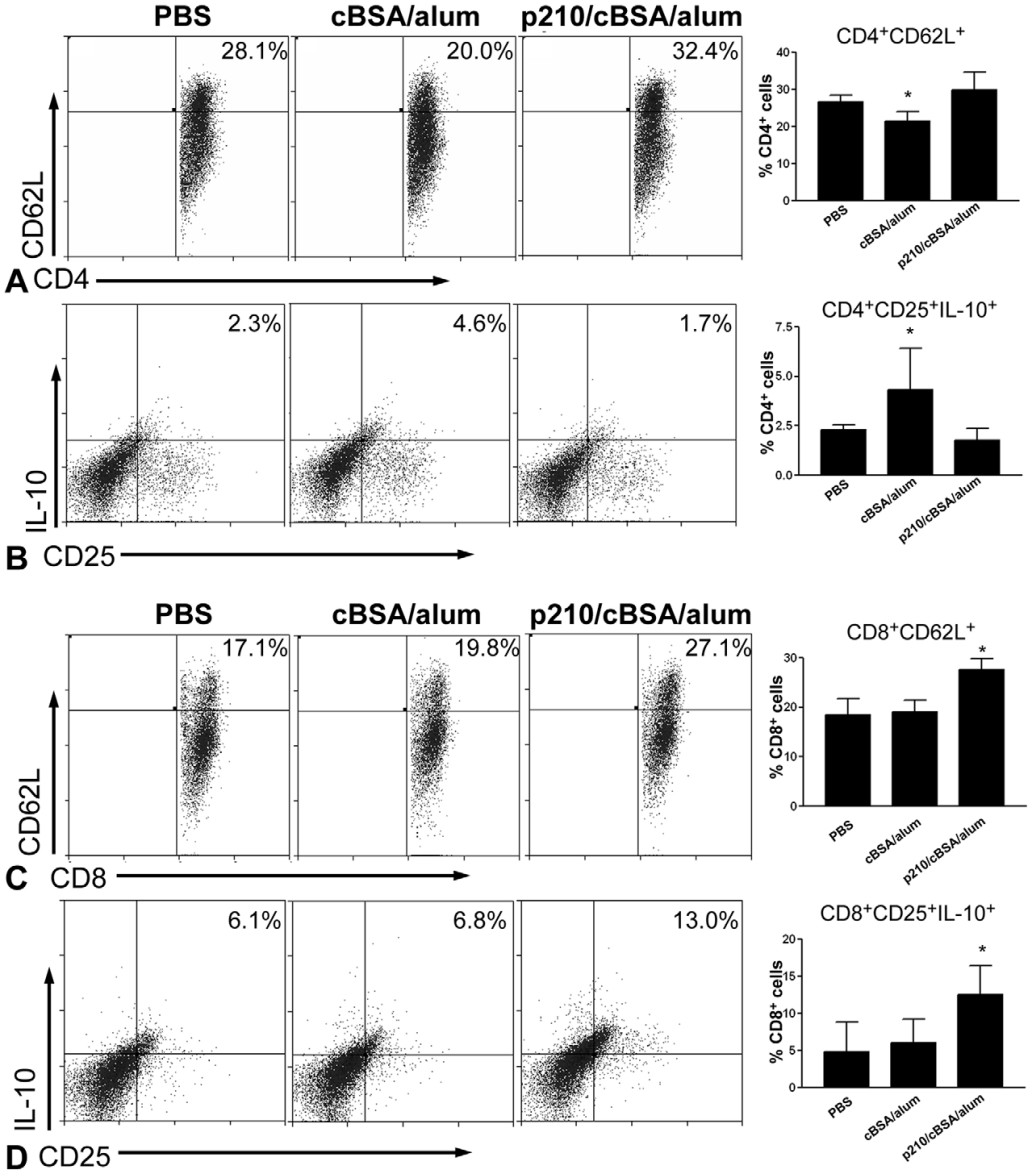
Spleen T lymphocyte response to p210 immunization. (A) Representative scatter plots of CD4^+^ gated cells expressing CD62L from spleens of the different groups, and the results plotted in a bar graph showed a significant reduction in the cBSA/alum group. (B) Representative scatter plots of CD4-gated CD25^+^IL-10^+^ cells, and the results plotted in a bar graph showed a significant increase in the cBSA/alum group that was attenuated in the p210/cBSA/alum group. (C) Representative scatter plots of CD8b^+^ gated cells expressing CD62L, and the results plotted in a bar graph showed a significant increase in the p210/cBSA/alum group. (D) Representative scatter plots of CD8b-gated CD25^+^IL-10^+^ cells, and the results plotted in a bar graph showed a significant increase in the p210/cBSA/alum group. For all graphs, *P<0.05 vs. other groups; N = 5 each; ANOVA followed by Newman-Keuls multiple group comparison.

One week after the primary immunization, splenic CD4^+^ T cells and CD4^+^CD69^+^ T cells were similar among the 3 groups (not shown), but CD4^+^CD62L^+^ T cells (Fig. 3A) were significantly reduced in cBSA/alum group compared to PBS and p210/cBSA/alum groups. This was coupled with a significantly increased CD4^+^CD25^+^IL-10^+^ T cell population in the cBSA/alum group. However, this increased response was significantly attenuated by p210/cBSA/alum immunization to levels comparable to PBS (Fig. 3B). Splenic CD4^+^CD25^+^IL-12^+^ T cells did not differ among the three groups (not shown). Thus, significant changes in the CD4^+^ T cell population occurred in the cBSA/alum group but not specific to the p210/cBSA/alum group.

#### CD8^+^ T cell response to immunization

At the same time-point, splenic CD8^+^ T cells and CD8^+^CD69^+^ T cells were similar among the 3 groups (not shown) but the CD8^+^CD62L^+^ T cell population was significantly increased in the p210/cBSA/alum group (Fig. 3C). This occurred as CD8^+^CD25^+^IL-10^+^ T cells in p210/cBSA/alum group was also significantly increased when compared to PBS or cBSA/alum groups (Fig. 3D). There were no differences in CD8^+^CD25^+^IL-12^+^ T cells among the 3 groups (not shown). CD8^+^CD25^+^ T cells were also similar among the 3 groups. Thus, p210 immunization elicited a CD8^+^ T cell response.

We also evaluated the percentage of CD4^+^ or CD8^+^ T cells and CD25 expression from mice euthanized at 13 or 25 weeks and observed no difference among the 3 groups specific to p210 immunization (not shown).

#### T cell response to p210 in vitro

T cell proliferation using CFSE was assessed in spleens of immunized WT mice stimulated with p210 three weeks after the second booster injection (Figure S1). WT mice were used to assure that potential antigenic exposure from serum cholesterol as confounding factors would be kept at a minimum given the severe levels of hypercholesterolemia in apoE-/- mice. Stimulation with p210 increased CD4^+^ T cell proliferation in both cBSA/alum and p210/cBSA/alum compared to PBS. CD8^+^ T cell proliferation was selectively highest in p210/cBSA/alum immunized mice compared to PBS and cBSA/alum.

### Effect of p210 immunization on dendritic cells

**Figure 4:**
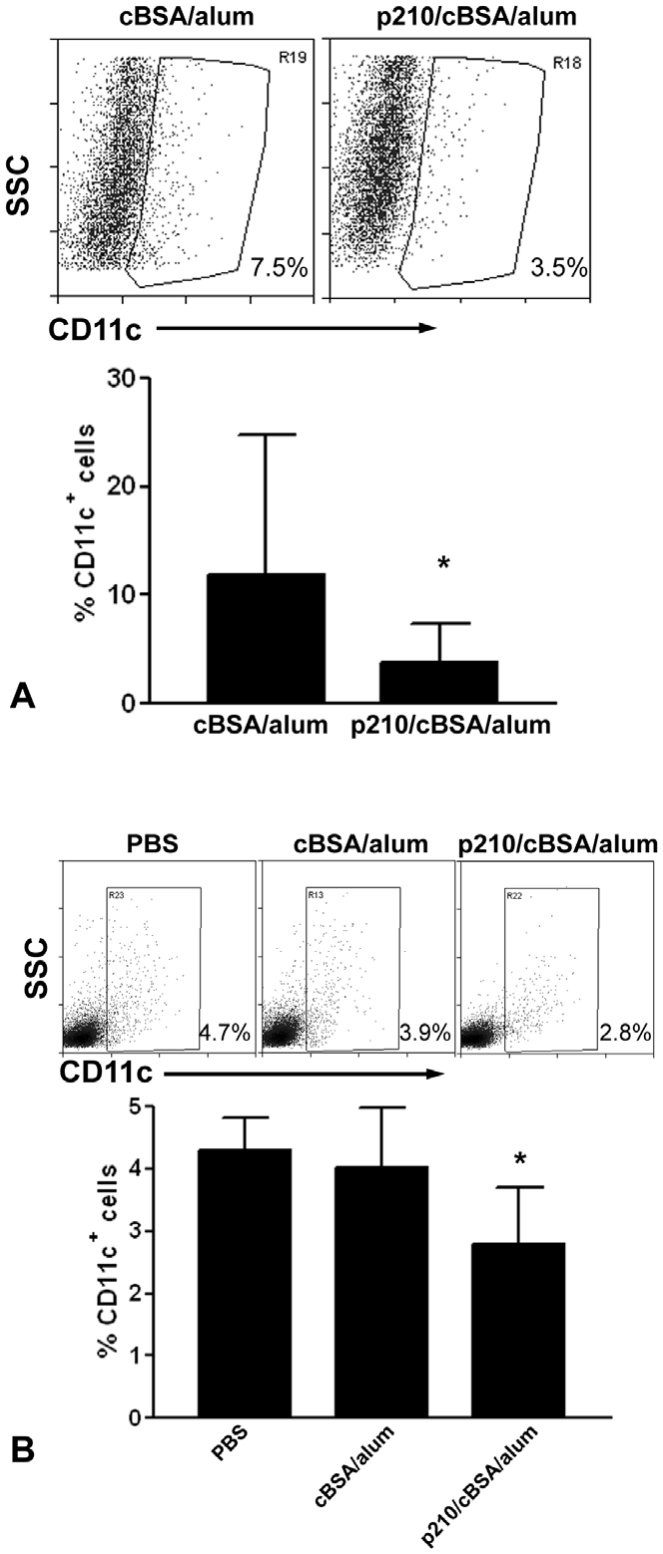
p210 immunization reduced DCs in vivo. Representative scatter plot of viable cells from the immunization site one week after primary immunization gated on CD11c (A, top panel). Mice that received PBS injection did not have palpable swelling and cell accumulation at the injection site, hence not included. Bar graph shows significantly reduced presence of CD11c^+^ DCs at the immunization site of the p210/cBSA/alum group (A, bottom panel; N = 10 each, *P<0.05 by Mann-Whitney test). (B) One week after the second booster injection, p210 immunized mice had significantly reduced CD11c^+^ DCs in pooled lymph nodes from bilateral axillary and cervical areas compared to cBSA/alum group (n = 5 in each group; P<0.05; ANOVA followed by Newman-Keuls multiple group comparison).

Since DCs were significantly reduced in the plaques of p210-immunized mice at euthanasia, DCs were assessed by flow cytometry at other sites at earlier time points. Cells from the subcutaneous immunization sites were isolated one week after the primary immunization or the second booster. The PBS group could not be included in this analysis because mice receiving PBS injection did not develop swelling or cell accumulation at the injection site. There were significantly fewer CD11c^+^ cells in p210/cBSA/alum group compared to cBSA/alum group at the immunization site 1 week after the primary immunization (Fig. 4A). CD11c^+^ cells in the lymph nodes were not different 1 week after the primary immunization (not shown), but were significantly reduced 1 week after the second booster in the p210/cBSA/alum group (Fig. 4B).

To determine if the DCs in the p210/cBSA/alum group changed in phenotype, CD86 expression was analyzed in the lymph nodes of mice 1 week after the second booster. There was a significant increase in the percentage of CD11c-gated CD86^−^ cells in the p210/cBSA/alum group, paralleled by a significant reduction in the percentage of CD86^+^ cells. However, CD86 mean fluorescent intensity among the groups did not change (Figure S2). The results indicate that immunization reduced the number of CD11c^+^CD86^+^ DCs but did not change the cell phenotype.

### Increased cytolytic activity of p210-immune CD8^+^ T cells against dendritic cells

**Figure 5:**
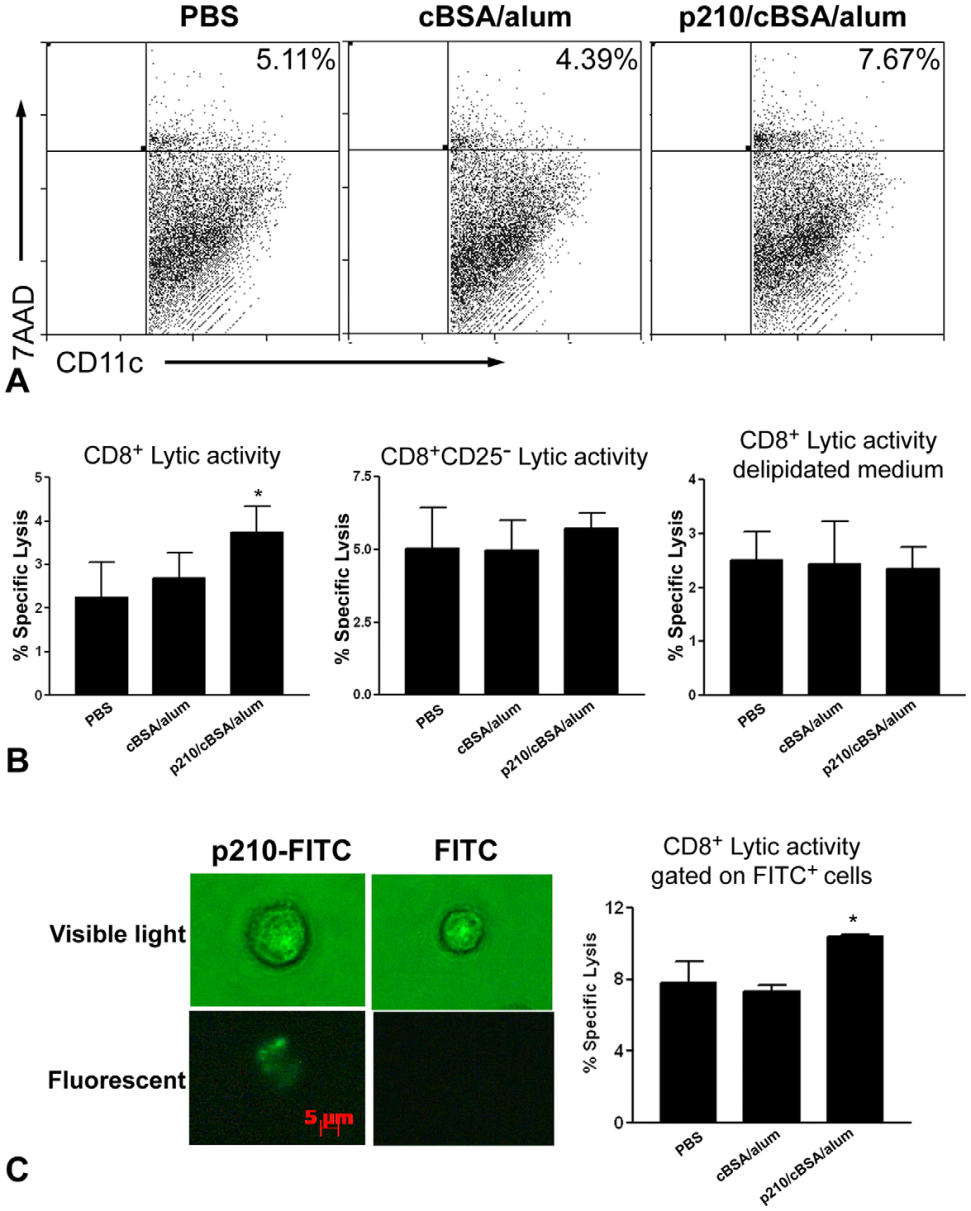
Increased cytolytic activity of CD8^+^ T cells from p210 immunized mice against dendritic cells in vitro. (A) Representative scatter plot of CD11c-gated cells stained with 7-AAD after co-culture for 4 hours with CD8^+^ T cells from the different experimental groups. CD8^+^ T cells from p210 immunized mice had a significantly higher cytolytic activity against DCs when compared to those from PBS or cBSA/alum groups (B, left panel; N = 11 each; *P<0.05). Depletion of CD25^+^ cells from the CD8^+^ T cells negated the lytic activity specific to p210 immunized mice (B, center panel; N = 4 each). Specificity of the increased lytic activity of CD8^+^ T cells from p210-immunized mice was also negated by performing the assay in media supplemented with 10% delipidated serum (B, right panel; N = 3 each). (C) Dendritic cells were able to uptake p210 as indicated by the presence of green fluorescence in the cell (left panel). When such dendritic cells were co-cultured with CD8^+^ T cells from p210-immunized mice, there was a significant increase of lytic activity when compared to those from PBS or cBSA/alum groups (right panel). Data were analyzed by ANOVA followed by Newman-Keuls multiple group comparison.

Given the observation that p210 immunization elicited a CD8^+^ T cell response and reduced DCs in the immunization sites, lymph nodes, and atherosclerotic plaques, we hypothesized that CD8^+^ T cells are the effector cells of the immune response with DCs as a potential target of lytic activity. To test this, we co-cultured syngeneic apoE (-/-) bone marrow derived DCs with CD8^+^ T cells from the various experimental groups. CD8^+^ T cells from p210 immunized mice significantly increased the percentage of DC lysis when compared to those from PBS or cBSA/alum groups (Fig. 5A and left panel of 5B).

Depletion of CD25^+^ cells from the CD8^+^ T cell pool negated the lytic activity specific to the p210/cBSA/alum group (Fig. 5B, middle panel) supporting the notion that CD8^+^CD25^+^ T cells are the effector cells of the response to p210 immunization.

The medium used to culture the cells is normally supplemented with 10% FBS, which contain apoB-100 on the LDL cholesterol fraction. To test if DC lysis by CD8^+^ T cells from the p210/cBSA/alum group is specific for the presence of lipid-associated antigens in the medium, the identical lytic assay was performed in 10% delipidated FBS. Specific lytic activity was lost when the assay was performed in delipidated FBS medium (Fig. 5B, right panel), supporting the notion that CD8^+^ T cells from the p210/cBSA/alum group specifically targeted lipid-associated antigens in serum.

### Lysis of p210-loaded dendritic cells by p210-immune CD8^+^ T cells

We further assessed lytic activity of CD8^+^ T cells from the different groups against p210-loaded target DCs. To assure that the peptide was taken up by the DCs, FITC-labeled p210 (p210-FITC, 100 µg/ml) was incubated with DCs for 18 hours and visualized using fluorescence microscopy. FITC was detected in DCs cultured with p210-FITC but not in cells incubated with FITC alone (Fig. 5C, bottom left panel). Visible light microscopy assured the FITC signal was localized to the DCs (Fig. 5C, top left panel). Lytic assay was then performed using p210-FITC loaded DCs as target cells. Lysis was assessed on FITC-gated DCs to assure that only p210-FITC loaded DCs were included in the analysis. Lytic activity was observed only in the DCs co-cultured with CD8^+^ T cells from the p210/cBSA/alum group (Fig. 5D).

### Adoptive transfer of p210-immune CD8^+^ T cells recapitulates the effect of p210 immunization

**Figure 6:**
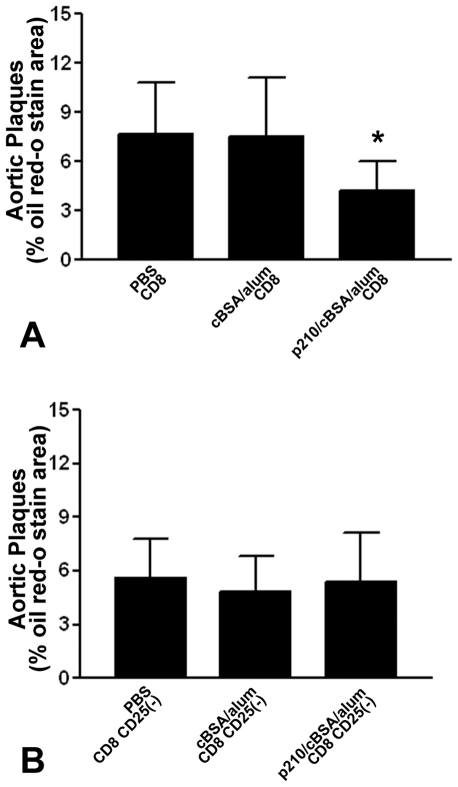
Adoptive transfer of CD8^+^ T cells from p210 immunized donors recapitulated the effect of p210 immunization. (A) CD8^+^ T cell recipient mice from p210/cBSA/alum immunized donors developed significantly smaller atherosclerotic lesions compared to the recipient mice of CD8^+^ T cells from other 2 groups (N = 9–10 each group; P<0.05). This effect was negated when CD25^+^ cells were depleted from the donor pool (B; 8–10 each group). Data were analyzed by ANOVA followed by Newman-Keuls multiple group comparison.

Donor CD8^+^T cells from PBS, cBSA/alum, or p210/cBSA/alum groups were adoptively transferred to 6–7 week-old naive recipient apoE (-/-) mice. At euthanasia, the recipient mice injected with CD8^+^ T cells from p210/cBSA/alum group developed significantly less atherosclerotic lesions in the aorta compared to the recipient mice injected with CD8^+^ T cells from PBS or cBSA/alum groups (Fig. 6A). The reduction in aortic lesions was coupled with decreased splenic CD11c^+^ DCs (PBS group: 4.3±1.7%; cBSA/alum group: 3.4±0.3%; p210/cBSA/alum group: 1.5±0.3%; n = 5 each group, P<0.05, p210/cBSA/alum group vs. PBS or cBSA/alum group). There was no significant difference in circulating levels of total cholesterol among 3 groups (PBS group: 1083±296 mg/dl; cBSA/alum group: 975±401 mg/dl; p210/cBSA/alum group: 1098±379 mg/dl). Aortic sinus plaque size was similar among the recipient groups. Macrophage presence was highest in recipients of PBS CD8^+^ T cells. There was significantly reduced CD4^+^ T cell presence in the aortic sinus of recipient mice injected with CD8^+^ T cells from p210/cBSA/alum donors compared to PBS or cBSA/alum group. No difference was observed in CD8^+^ T cells in the aortic sinus (Figure S3).

Depletion of CD8^+^CD25^+^ T cells nullified the reduction in atherosclerosis observed in the recipient mice injected with CD8^+^ T cells from p210/cBSA/alum group (Fig. 6B), supporting the notion that the functional T cell population induced by immunization is specific to CD8^+^CD25^+^ T cells. There was also a lack of difference in CD11c^+^ DCs among the recipient groups after CD8^+^CD25^+^ depletion (PBS group: 2.9±0.8%; cBSA/alum group: 3.1±1.2%; p210/cBSA/alum group: 2.3±0.4%; n = 8–10 each group).

Adoptive transfer of B cells isolated from the spleens of p210 immunized donor mice did not affect atherosclerosis in recipient mice compared to mice receiving B cells from other donors (Figure S4A). To assess CD4^+^CD25^+^ T cells as possible athero-protective mediators induced by sub-cutaneous p210 immunization, we adoptively transferred CD4^+^CD25^+^T cells into naïve recipient apoE (-/-) mice. There was no difference in lesion size among the 3 groups of CD4^+^CD25^+^ T cell recipients at a dose of 1×10^5^ cells/mouse or 3×10^5^ cells/mouse (Figure S4B and C, respectively).

### Immunization with p210 does not affect the adaptive immune response to other T cell dependent or independent antigens

**Figure 7:**
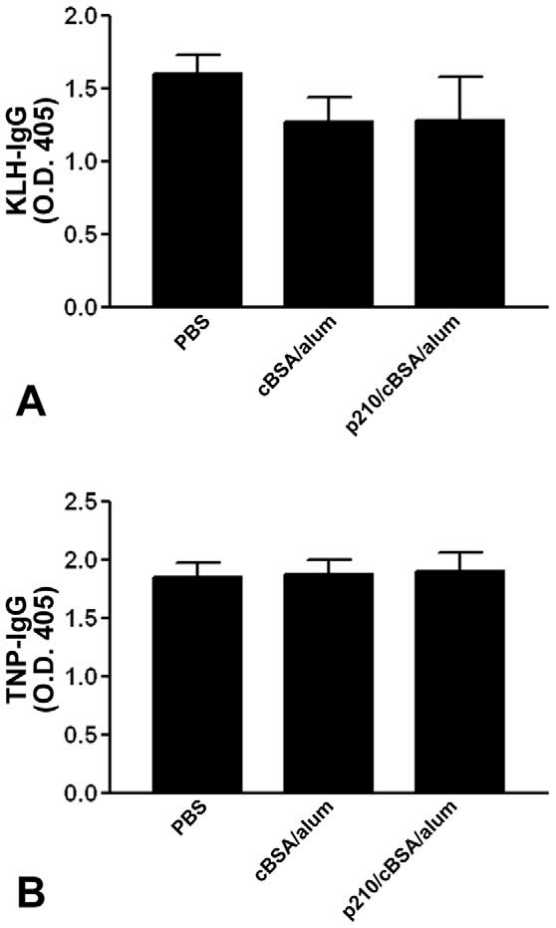
IgG titers against KLH or TNP after p210 immunization. (A) Prior immunization with p210 did not affect the efficacy of subsequent T cell dependent (KLH, n = 3–6 each group) or (B) T-cell independent (TNP, n = 4–5 each group) immunization as assessed by the IgG antibody titers when compared to mice received PBS or cBSA/alum. Data were analyzed by ANOVA followed by Newman-Keuls multiple group comparison.

Given the observations that p210 immunization decreased CD11c^+^ DCs and reduced adaptive IgG response to p210, we next tested if such modulation of DCs by p210 immunization would alter the host immune response to other antigens. KLH was chosen as a prototypical T cell dependent and TNP as a T cell independent antigen. We first immunized mice with p210 as described in previous sections followed by two separate subcutaneous KLH immunizations or intra-peritoneal injection of TNP-LPS. Using the KLH- or TNP-IgG titer as a surrogate for the efficacy of individual immunization, we found that there was no difference in KLH- or TNP-IgG titers (Fig. 7A and B, respectively) between p210 immunized mice and the titers from mice of PBS or cBSA/alum groups.

## Discussion

In this study, we showed that: (a) subcutaneous p210 immunization elicited a functional CD8^+^ T cell immune response; (b) p210 immunization reduces DCs at the immunization sites and in atherosclerotic plaques; (c) the athero-protective effect of subcutaneous p210 immunization is mediated by CD8^+^ T cells but not by CD4^+^CD25^+^ T cells or B cells; and (d) DCs are potential targets of cytolytic CD8^+^ T cells elicited by p210 immunization.

There have been 4 prior publications describing the athero-protective effect of p210 immunization [4], [6]–[8], but there was no definitive report regarding the specific cellular immune responses responsible for such effect of p210 immunization. The two studies by Fredrikson et al. did not delineate the responsible cellular mechanism [4], [6]; whereas Wigren et al. suggested that Tregs are involved in the response but did not test in vivo the specific cell type proposed [8]. The study by Klingenberg et al. described intra-nasal p210 immunization increased splenic CD4^+^IL-10^+^ T cells in p210 group when compared to untreated control group but did not define further the role of splenic CD4^+^IL-10^+^ T cells in mediating the effect of intra-nasal p210 immunization [7]. However, the site of antigenic entry, intra-nasal in their study compared to subcutaneous in our study, will likely yield different results. The current study systematically delineates the immune responses after subcutaneous p210 immunization and identifies CD8^+^ T cells as the immune cell type that mediates the protective effect against atherosclerosis. We further show that targeting DCs is a potential immune mechanism underlying the protective effect of immunization with p210 against atherosclerosis.

Active immunization with homologous LDL or apoB-100 related peptides reduces atherosclerotic lesions in experimental animals [3], [4], [9]–[14]. Antibodies elicited by such immunization had been postulated to be responsible for the athero-protective effect [13], [14]. On the other hand, significant reduction of atherosclerosis after immunization with native LDL did not depend on high titers of antibodies against oxidation–specific epitopes in LDL [11]. We observed that mice immunized with cBSA/alum developed higher p210 IgG titers than mice immunized with p210/cBSA/alum and the transfer of B cells from p210/cBSA/alum immunized donors failed to reduce atheosclerosis in recipient mice. This is in agreement with a previous study where p210 immunization resulted in reduced p210 antibody titers compared with the carrier group [6]. Thus, our results combined with those from the previous studies strongly suggest that the humoral immune response is not likely to be responsible for the athero-protection elicited by p210 immunization. Our results also suggest the following points: (a) the presence of elevated p210 IgM titers in all 3 groups of mice suggests that atherosclerosis is associated with an endogenous p210 humoral immune response; (b) CD8^+^ T cell response and p210 IgG titer could be used as markers for an immune response to p210 but only CD8^+^ T cell response would be an immune marker for efficacy against atherosclerosis.

LDL immunization activates both CD4^+^ and CD8^+^ T cells [13]. However LDL immunization was effective against atherosclerosis even in CD4/apoE double knockout mice [15], suggesting CD4^+^ T cells are not important mediators of the beneficial effect of LDL immunization. CD4^+^ T cells are pro-atherogenic as evidenced by the aggravation of atherosclerotic lesions in mice receiving naïve or antigen-primed CD4^+^ T cells [16], [17]. Interestingly, the CD8^+^ T cell population increased significantly in CD4/apoE double knockout mice compared to apoE (-/-) mice [15] but the role of CD8^+^ T cells in mediating the effect of LDL immunization has not been previously addressed.

A subset of CD4^+^ T cells, CD4^+^CD25^+^ T cells, reduce atherosclerotic lesions after being adoptively transferred into recipient mice [18], [19]. The recent reports [7], [8] on the possible role of CD4^+^CD25^+^ T cells in mediating the reduction in p210 immunized mice prompted us to check its role as well. However, CD4^+^CD25^+^ T cells are not likely to be responsible for the beneficial effects of p210 immunization since the CD4^+^CD25^+^ T cell recipient group did not recapitulate the athero-protective effect selective to p210 immunization at the 2 doses tested.

CD8^+^ T cells exist in atherosclerotic plaques [20], [21] but their role in atherogenesis has not been defined. The observation that atherosclerotic lesions in MHC-I deficient mice were significantly larger than those in wild type mice suggested a possible role of MHC-I and CD8^+^ T cells in atherogenesis [22]. However direct experimental proof has been lacking. Another recent report suggested early activation of CD8^+^ T cells induced by hypercholesterolemia without delineating specific mechanistic insight [23]. An important finding of our study is that p210 immunization elicited a prominent CD8^+^ T cell response, its significance highlighted by the reduction of atherosclerosis in mice receiving CD8^+^ T cells from the p210/cBSA/alum immunized donor group. These observations clearly define the specific role of CD8^+^ T cells in mediating the athero-protective effect of p210 immunization and suggest the possible use of CD8^+^ T cell response as a potential marker for the efficacy of p210 immunization. Negation of the reduction in atherosclerosis specific to p210 after depletion of CD25^+^ cells from the donor CD8^+^ T cells further suggest that the CD8^+^CD25^+^ T cell population is the effector cell involved in reducing atherosclerosis. In another arterial injury model, we recently reported that CD8^+^ T cells are activated after arterial injury and are the specific effectors in modulating neointimal formation [24]. Taken together, these reports suggest that, in addition to CD4^+^ T cells, CD8^+^ T cells also play an important role in modulating arterial responses to various injury stimulations.

Dendritic cells are present in atherosclerotic plaques [25], [26] and many studies have suggested a pivotal role of DCs in atherogenesis. Intimal DCs rapidly ingest lipid and become foam cells when mice are fed hyper-cholesterolemic diet. Depletion of these intimal DCs reduces foam cell formation [27]. Deficiency of the chemokine receptor CX3CR1, a regulator of monocyte adhesion and migration, decreases DC accumulation in the arterial wall leading to reduced atherosclerosis [28]. Deficiency of CD11c, a surface marker on DCs, reduced atherosclerotic lesions as well [29]. Our result showing reduced DCs in the p210-immunization site, lymph nodes, and plaques suggest that DC is one of the potential targets of CD8^+^ T cell mediated athero-protective effect of p210 immunization.

The CD8^+^ T cells from p210 immunized mice preferentially increased its cytolytic function compared with those from PBS or cBSA/alum immunized mice against identical DC targets. The results clearly establish that p210 immunization specifically altered CD8^+^ T cell function. What remains unclear is how DCs are targeted by p210 primed CD8^+^ T cells. Results from our in vitro experiments and several prior publications may provide some insights in this matter. Because p210 is derived from apoB-100, the cytolytic response is likely against apoB-100 fragments displayed on MHC class I of the target DCs. The only source of apoB-100 in our in vitro experiments is the fetal bovine serum (FBS) used for culture. ApoB-100 in FBS of culture media has been the subject of investigation by researchers confounded by the immune response observed in recipients of cell-therapy treatments [30]. Both experimental and clinical studies showed that the immune system reacted against the second set of cell therapy injections and elicited both antibody responses (implicating MHC class II) and cytolytic responses (implicating MHC class I). Exogenous antigens are usually processed by the MHC class II pathway. However, antigen cross-presentation is a well-accepted alternative in endosomal processing of antigens. Interestingly, mononuclear cells differentiating into DCs increase surface expression of the LDL receptor, which mediates antigen presentation [31] making the LDL receptor at least one of possible pathways for uptake. We therefore speculated that some of the LDL-cholesterol in the FBS would be taken up by DCs, get processed through the endosomal system and subsequently be cross-presented to MHC class I and displayed on the surface of the DCs, thus being recognized and targeted by p210 primed CD8^+^ T cells. Extending the notion that apoB-100 presented by DCs is the antigenic target in our study, removal of the source of apoB-100 in our in vitro experiments, the LDL in FBS, would predictably reduce the specific cytolytic activity of CD8^+^ T cell from the p210/cBSA/A mice. Our results indeed show that performing the lytic assay in delipidated serum medium had this outcome. Loading of target DCs with FITC-labeled p210 further confirmed the lytic activity specific to CD8^+^ T cell from the p210/cBSA/A mice. However at this time we do not know how p210 is processed in DCs for MHC-I presentation and the exact sequence of p210 motif on MHC-I molecule. Considering the fact that DCs are the main cell type that process and present antigens, another predictable outcome of the targeted lysis of DCs in vivo would be reduced humoral immune response to the p210 antigen. Again, the reduced antibody response in the p210 immunized group compared with the cBSA group is compatible with this notion.

Whether the reduction of DCs after p210 immunization alters the immune responses to other antigens was also investigated. This has an important clinical implication because human subjects receive multiple vaccinations throughout their life-span. The IgG titer against KLH (a T cell depenent antigen), or TNP (a T cell indepenent antigen) post p210 immunization did not differ among 3 groups. Given the intimate interaction among cellular and humoral immune components for antibody production, this intact antibody response suggests that the process of antigen recognition and presentation by DCs, cooperation between the antigen-specific B and T lymphocytes via various co-stimulatory molecules and cytokines, isotype switching and cellular migration among lymphoid organs remain intact in the immune response to unrelated antigens after p210 immunization.

In conclusion, we have shown that immunization with native p210 in vivo reduced DCs at the immunization sites and in the atherosclerotic plaques, reduced macrophage infiltration in the plaques, and decreased aortic atherosclerosis. More importantly we show that the athero-protective effect of p210 immunization is mediated by CD8^+^ T cells. Our results not only provide additional experimental evidence to support the concept of active immunization using apoB-100 related peptides to modulate atherosclerosis but also delineate the cell type that mediates such effect.

## Materials and Methods

### Selection of peptides and their preparation for immunization

The establishment and screening of human apoB-100 peptides has been reported [32]. Based on our pilot experiments and prior reports [33], [34], we selected peptide 210 (p210, KTTKQ SFDLS VKAQY KKNKH) as a candidate immunogen. Native p210 peptide (Euro-Diagnostica AB, Sweden) was conjugated to cationic bovine serum albumin (cBSA) as carrier using a method described previously [3], [4]. Alum was used as adjuvant and mixed with peptide/cBSA conjugate with 1∶1 ratio in volume. Peptide conjugation and mixing with alum were freshly prepared prior to each immunization.

### Immunization protocols

Male apoE (-/-) mice (Jackson Laboratories) were housed in a pathogen-free animal facility accredited by the Association for the Assessment and Accreditation of Laboratory Animal Care International and kept on a 12-hour day/night cycle with unrestricted access to water and food. In a pilot experiment, p210 immunization using 100 µg dose conferred optimum athero-reduction compared to 25 or 50 µg dose. Hence 100 µg dose was used for all subsequent experiments. The Institutional Animal Care and Use Committee of Cedars-Sinai Medical Center approved the experimental protocols (IACUC No. 1248, 1563 and 2567).

Mice, maintained on normal chow diet, received subcutaneous primary immunization in the dorsal area between scapulas at 6–7 weeks of age, followed by a booster at 9 and 12 weeks of age. One week after last booster, diet was switched to high cholesterol chow (TD 88137, Harlan-Teklad) and continued until euthanasia at the age of 25 weeks. Separate groups of mice receiving PBS or cBSA/alum at the same immunization time-points served as control. Some mice were sacrificed at 8 or 13 weeks of age to assess immune response against p210.

### Tissue harvesting and preparation

At euthanasia the hearts were harvested and embedded in OCT compound (Tissue-Tek) for cryo-section. Whole aortas were cleaned, processed and stained with Oil-red-o to assess the extent of atherosclerosis *en face* with computer-assisted histomorphometry [3], [4].

### Immunohistochemistry and histomorphometry

The sections from aortic sinus were stained with MOMA-2 (Serotec), CD11c, CD4, or CD8b (eBioscience) antibody to identify macrophages, dendritic cells, or T cells immunohistochemically using standard protocol. Oil-red-o stain for plaque size was done using standard protocol. Computer-assisted morphometric analysis was performed on samples blinded to the person to assess histomorphometry as described previously [3], [4]. The person who performed the computer-assisted analysis was blinded toward to the treatment of the mice.

### Serum ELISA

Flat-bottomed 96-well polystyrene plates (MaxiSorp, Germany) were pre-coated with 100 ul (20 µg/ml) P210, KLH, TNP-KLH (Biosearch Technologies T-5060) or BSA (2 µg/ml for IgG or 10 µg/ml for IgM) respectively by incubation overnight at 4°C to assess antibody levels using standard protocol. The coating concentration and serum dilution was optimized in pilot experiments. Goat anti-mouse HRP -IgG (Pierce 31437) or IgM (Southern Biotech) were used as detecting antibodies and the bound antibodies were detected by developing in ABTS (Southern Biotech) as substrate and optical density values were recorded at 405 nm.

### Flow cytometry

Flow cytometry was performed using standard protocols with antibodies listed in Table 2 and a FACScan (Becton Dickinson) or a CyAn ADP analyzer (Beckman Coulter). For intracellular cytokine staining, Brefeldin A (3 µg/ml) was added to the cultured cells for 2 hours before cells were subject to staining procedure. Cell membranes were permeabilized for staining intracellular molecules.

**Table 2:** Antibodies used for flow cytometry analysis.

Antigen	Clone	Type	Supplier
CD4	GK1.5	FITC-Rat IgG2b, κ	BD Pharmingen
CD8b.2	53-5.8	FITC-Rat IgG1, κ	BD Pharmingen
CD25	PC61.5	PE-Rat IgG1, λ	eBioscience
IL-10	JES5-16E3	Percp-Cy5.5- Rat IgG2a, κ	eBioscience
IL-12	Clone C17.8	Percp-Cy5.5- Rat IgG2b, κ	eBioscience
CD11c	HL3	FITC-Hamster IgG1, λ	BD Pharmingen
CD86	GL1	PE-Rat IgG2a, κ	BD Pharmingen
CD62L	MEL-14	PE-Rat IgG2a, κ	eBioscience
CD69	H1.2F3	APC American hamster IgG	eBioscience

### Adoptive transfer experiment

Male apoE (-/-) mice on regular chow received subcutaneous immunization as described in previous paragraph and were sacrificed at 13 weeks of age as donors. Splenocytes from the same treatment group were pooled before cell isolation. Donor CD8^+^ T cells, CD4^+^CD25^+^ T cells or B cells were isolated using Dynabeads FlowComp (Invitrogen) according to the manufacturer's protocols. CD4^+^ T cells were negatively selected from the splenocytes followed by positive selection of CD4^+^CD25^+^ cells. B cells were negatively isolated whereas CD8^+^ T cells were positively isolated first and released from beads. The purity of pooled CD8^+^ T cells, CD4^+^CD25^+^ T cells and B cells was ∼90%. The isolated CD8^+^ T-cells (1×10^6^ cells/mouse), CD4^+^CD25^+^ T-cells (1×10^5^ or 3×10^5^ cells/mouse) or B cells (2×10^7^ cells/mouse) were then adoptively transferred to naïve male apoE (-/-) recipient mice at 6–7 weeks of age via tail vein injection.

For B cell transfer, the number of 2×10^7^ cells/mouse was chosen based on two prior reports [35], [36]. For CD4^+^CD25^+^ T cells transfer, we chose 2 doses for our experiment based on published literature [18], [19], [37]. For CD8^+^ T-cells, 1×10^6^ cells was chosen based on a report from the field of autoimmune disease [38]. We did not adoptively transfer CD4^+^ T cells because naïve or antigen-primed CD4^+^ T cells are known to be pro-atherogenic [16], [17].

Recipient mice were fed normal chow until 13 weeks of age when chow was switched to high cholesterol diet until euthanasia at 25 weeks of age. Aortas were harvested to assess the extent of atherosclerosis.

### KLH or Trinitrophenyl-lipopolysaccharide (TNP-LPS) Immunization

KLH was chosen as a prototypical T cell dependent and TNP as a T cell independent antigen. Male C57/BL6 mice on regular chow received subcutaneous immunization with p210 conjugate or adjuvant control as described in previous paragraphs for apoE (-/-) mice. At 13 and 15 weeks of age mice were subcutaneously immunized with 100 µg KLH (with alum as adjuvant) at injection sites away from p210 sites or injected intraperitoneally with 100 µg TNP-LPS (Sigma). KLH or TNP immunization was done in separate groups of mice. Blood was collected via retro-orbital puncture at euthanasia (16 weeks of age).

### In vitro experiment

#### T cell proliferation assay

Male C57/BL6 mice fed regular chow were immunized as detailed above for the apoE-/- mice. WT mice were used to assure that potential antigenic exposure from serum cholesterol as a confounding factor would be kept at a minimum given the severe levels of hypercholesterolemia in apoE-/- mice. At 15 weeks of age, 3 weeks after the second booster, the mice were euthanized and the spleens harvested for splenocyte isolation. Following RBC lysis, splenocytes pooled from 2 mice per group were labeled with CFSE (2 µM at 37°C for 10 minutes). Equal numbers of cells were seeded into culture dishes and the cells were stimulated with 50 µg/ml p210 in complete medium (RPMI-1640 supplemented with 10 U/ml IL-2). Cells were harvested after 5 days and stained with fluorescent anti-CD4 or CD8b antibody and subjected to flow cytometry.

#### Generation of BM-derived dendritic cells (BMDCs)

The method for generating BMDC with GM-CSF was adapted from a previous publication with modification [39]. Briefly, bone marrow cells from femurs and tibiae of male apoE (-/-) mice were plated into 10 cm culture plates (Falcon) with 20 ml complete RPMI-1640 containing 10 ng/ml GM-CSF (R&D Systems) and 10 ng/ml IL-4 (Invitrogen). Cells were washed and fed on day 3 and day 5 by removing the old medium followed by replenishing with fresh culture medium with GM-CSF and IL-4. On day 8, the immature DC appeared as non-adherent cells under the microscope and harvested by vigorous pipetting and subcultured into new culture plates with 2×10^5^ DCs in 1.5 ml medium.

#### CD8^+^ T cell isolation and co-culture with dendritic cells

Donor male apoE (-/-) mice for CD8^+^ T cells were immunized with PBS, cBSA/Alum, or cBSA/Alum/P210 according to the schedule described in earlier paragraphs and splenocytes were harvested at 13 weeks of age. CD8^+^ T cells were negatively isolated using a CD8 selection Dynabeads kit (Invitrogen) per manufacturer's protocol. The selected CD8^+^ T-cells were then co-culture with DCs in a CD8∶DC ratio of 3∶1. A series of pilot studies has been performed to determine the optimal CD8∶DC ratio for this assay. After co-culture for 4 hours, cells were collected and processed for flow cytometric determination of CD11c and 7-AAD by LSR II flow cytometer (BD Biosciences) and data was analyzed with Summit V4.3 software. An additional lytic assay was developed wherein BMDCs were transferred into culture medium with 10% delipidated FBS (Cocalico Biologicals Inc.) overnight prior to CD8 co-culture and the lytic assay performed in 10% delipidated FBS medium. Lytic assay using p210-loaded DCs was designed with FITC-labeled p210 using a commercially available FITC labeling kit (Thermo Scientific). BMDCs were loaded with FITC-labeled p210 (100 µg/ml) for 18 hours, determined by pilot experiments to be optimal for maximal uptake. The cells were then washed and co-cultured with CD8^+^ T cells as described above for 4 hours. Lysis was then assessed on FITC-gated DCs to assure that only p210-FITC antigen-loaded DCs were included in the analysis. Dendritic cell death without CD8^+^ T cells in the co-culture was used as baseline and percentage of specific lysis of cells was calculated using a method described previously [40].

### Statistics

Data are presented as mean ± SD. Number of animals in each group is listed in text or figure legend. Data were analyzed by ANOVA followed by Newman-Keuls multiple group comparison, or by t-test when appropriate. P<0.05 was considered as statistically significant.

## Supporting Information

Figure S1 **T lymphocyte proliferation in response to p210 stimulation.** Histogram depicting cell proliferation of CFSE-labeled splenocytes from the immunized groups in response to stimulation with p210 (50 µg/ml). Stimulation with p210 increased CD4^+^ T cell proliferation in both cBSA/alum (49.5%) and p210/cBSA/alum (56.6%) compared to PBS (32.2%). CD8^+^ T cell proliferation was highest in p210/cBSA/alum immunized mice (40.1%) compared to PBS (16.7%) and cBSA/alum (25.8%).
(TIF)

Figure S2 **Lymph node dendritic cell CD86 expression one week after second booster.** Significant increase in the percentage of CD11c-gated CD86^−^ cells (A) in the p210/cBSA/alum group compared to PBS and cBSA/alum. Concomitant decrease in CD11c-gated CD86^+^ cells (B) in the p210/cBSA/alum group compared to PBS and cBSA/alum. No significant difference was observed in the CD86 mean fluorescent intensity (MFI) among the groups (C). *p<0.05; N = 5 each group.
(TIF)

Figure S3 **Aortic sinus phenotype of CD8^+^ T cell recipient mice.** Aortic sinus plaque size was similar among the recipient groups (A). Macrophage infiltration assessed by MOMA-2 stain (B) was significantly reduced in both cBSA/alum CD8^+^ T cell and p210/cBSA/alum CD8^+^ T cell recipient groups. CD4^+^ T cells were significantly reduced in recipient mice injected with CD8^+^ T cells from p210/cBSA/alum donors compared to PBS or cBSA/alum group (C). No difference was observed in CD8^+^ T cells in the aortic sinus (D). *p<0.05 vs. PBS; †p<0.05 vs. cBSA/alum; N = 6–9 each group.
(TIF)

Figure S4 **Adoptive transfer of B cells or CD4^+^CD25^+^ T cells from immunized donor groups.** Aortic atherosclerosis was not significantly different among the recipients of B cells (A), or CD4^+^CD25^+^ T cells at a dose of 1×10^5^ cells/mouse (B) or 3×10^5^ cells/mouse (C) adoptively transferred from donor mice of the different immunized groups into naïve mice. N = 9–13 each group.
(TIF)
