# Supplementary figures and images for: CD8+ T Cells Mediate the Athero-Protective Effect of Immunization with an ApoB-100 Peptide
Source: PLoS One. 2012 Feb 9;7(2):e30780. doi: 10.1371/journal.pone.0030780 (PMC3276497; doi:10.1371/journal.pone.0030780)

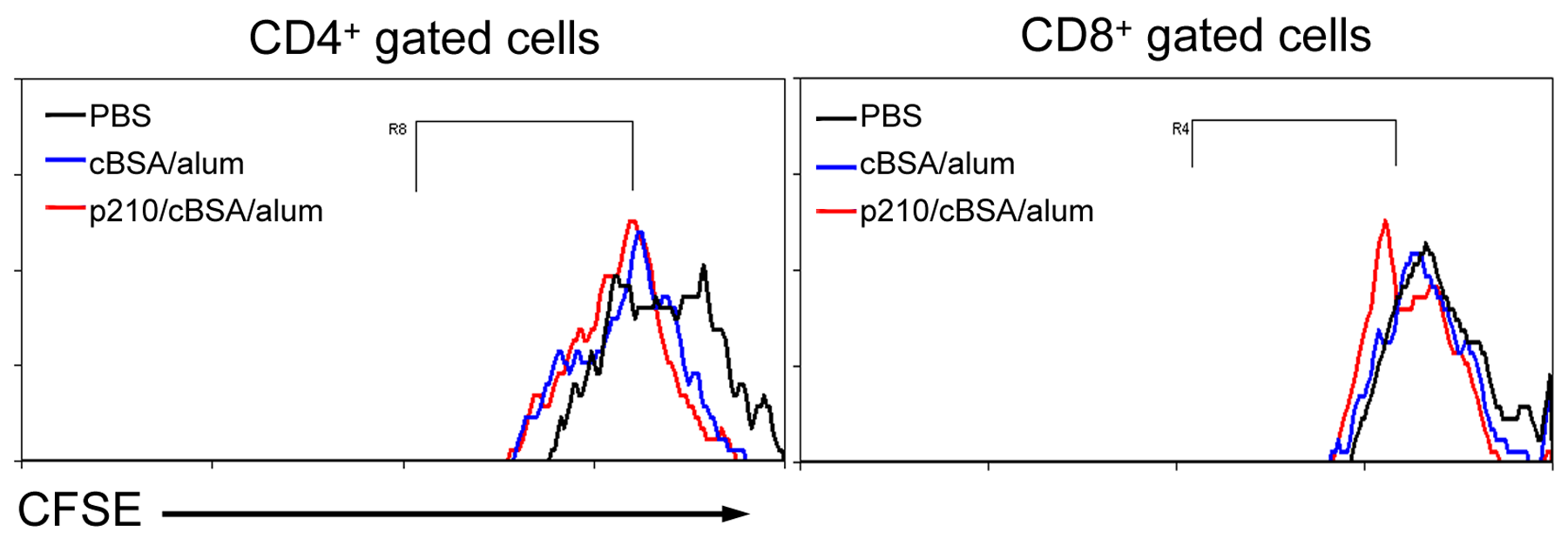

Supplement: Figure S1 — T lymphocyte proliferation in response to p210 stimulation. Histogram depicting cell proliferation of CFSE-labeled splenocytes from the immunized groups in response to stimulation with p210 (50 µg/ml). Stimulation with p210 increased CD4+ T cell proliferation in both cBSA/alum (49.5%) and p210/cBSA/alum (56.6%) compared to PBS (32.2%). CD8+ T cell proliferation was highest in p210/cBSA/alum immunized mice (40.1%) compared to PBS (16.7%) and cBSA/alum (25.8%). (TIF) [file pone.0030780.s001.tif]

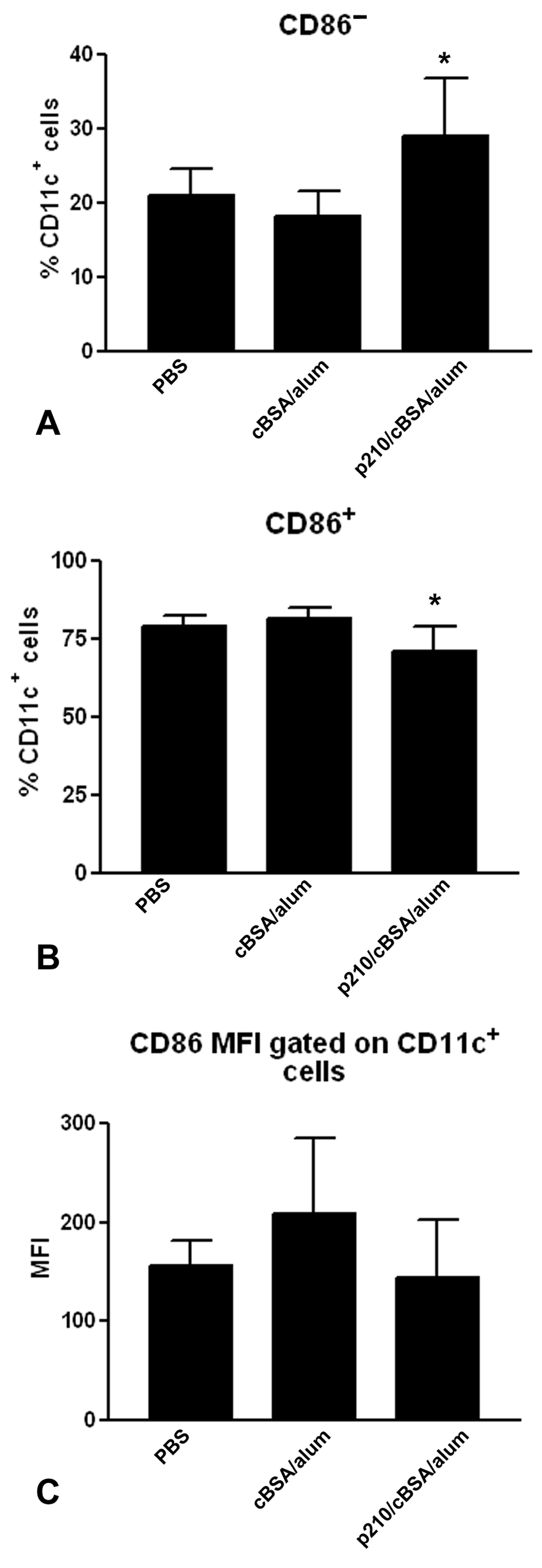

Supplement: Figure S2 — Lymph node dendritic cell CD86 expression one week after second booster. Significant increase in the percentage of CD11c-gated CD86− cells (A) in the p210/cBSA/alum group compared to PBS and cBSA/alum. Concomitant decrease in CD11c-gated CD86+ cells (B) in the p210/cBSA/alum group compared to PBS and cBSA/alum. No significant difference was observed in the CD86 mean fluorescent intensity (MFI) among the groups (C). *p<0.05; N = 5 each group. (TIF) [file pone.0030780.s002.tif]

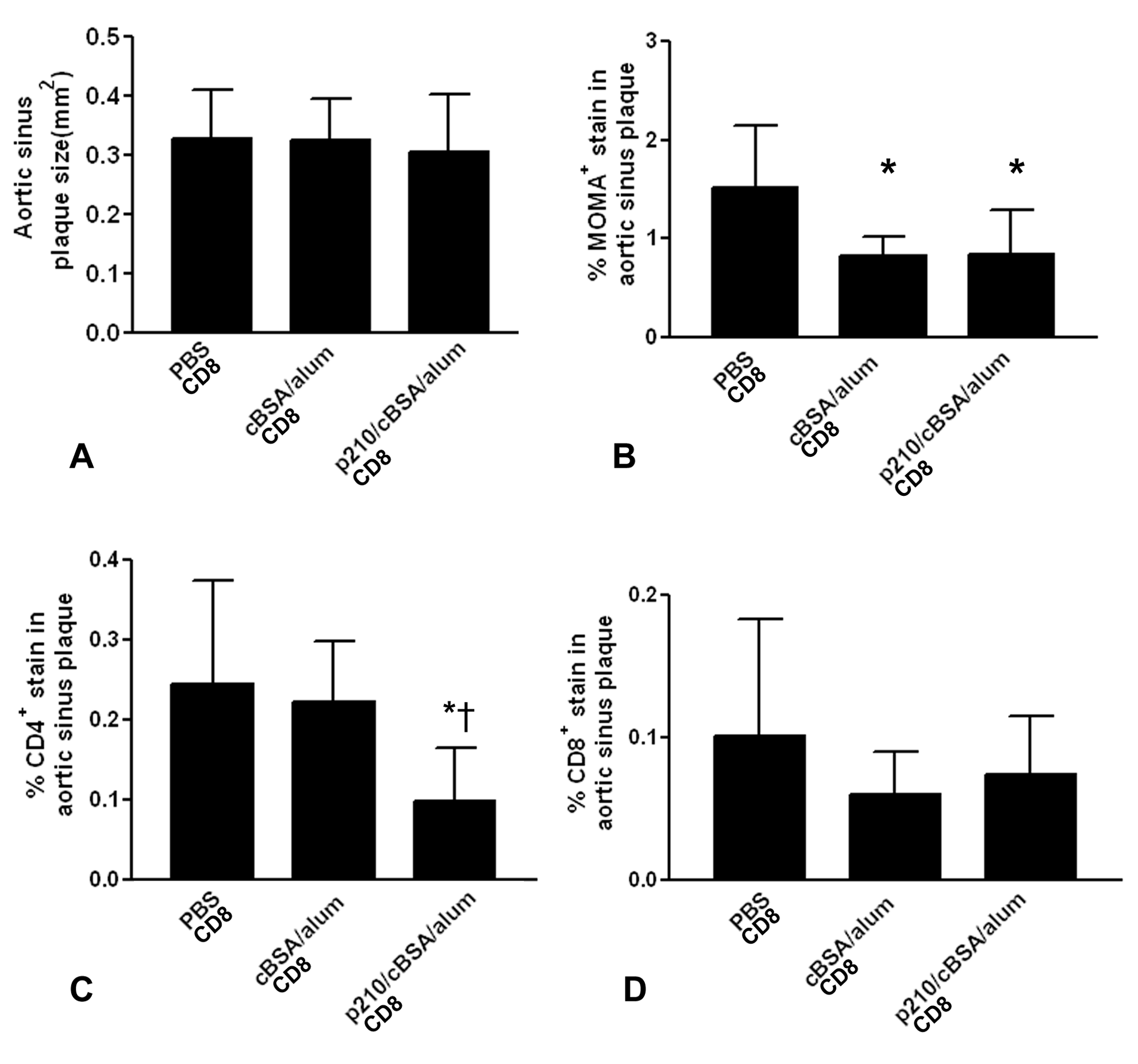

Supplement: Figure S3 — Aortic sinus phenotype of CD8+ T cell recipient mice. Aortic sinus plaque size was similar among the recipient groups (A). Macrophage infiltration assessed by MOMA-2 stain (B) was significantly reduced in both cBSA/alum CD8+ T cell and p210/cBSA/alum CD8+ T cell recipient groups. CD4+ T cells were significantly reduced in recipient mice injected with CD8+ T cells from p210/cBSA/alum donors compared to PBS or cBSA/alum group (C). No difference was observed in CD8+ T cells in the aortic sinus (D). *p<0.05 vs. PBS; †p<0.05 vs. cBSA/alum; N = 6–9 each group. (TIF) [file pone.0030780.s003.tif]

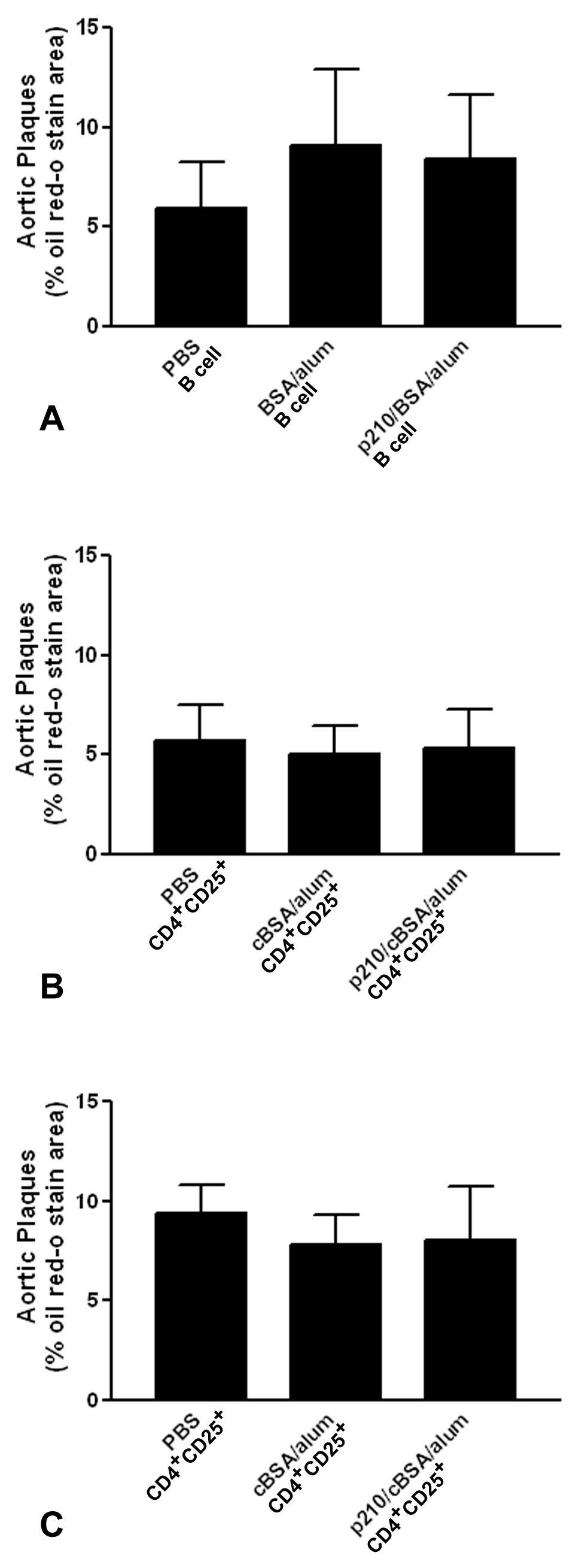

Supplement: Figure S4 — Adoptive transfer of B cells or CD4+CD25+ T cells from immunized donor groups. Aortic atherosclerosis was not significantly different among the recipients of B cells (A), or CD4+CD25+ T cells at a dose of 1×10^5^ cells/mouse (B) or 3×10^5^ cells/mouse (C) adoptively transferred from donor mice of the different immunized groups into naïve mice. N = 9–13 each group. (TIF) [file pone.0030780.s004.tif]
